# Syndrome of inappropriate antidiuretic hormone secretion in a patient with colon cancer using CAPOX plus bevacizumab therapy: a case report

**DOI:** 10.1186/s40780-025-00476-9

**Published:** 2025-07-31

**Authors:** Masanobu Uchiyama, Takafumi Inoue, Daibo Kojima, Masato Watanabe, Motoyasu Miyazaki, Takafumi Nakano, Takuya Yamashina, Osamu Imakyure, Koichi Matsuo

**Affiliations:** 1 Department of Oncology and Infectious Disease Pharmacy, Faculty of Pharmaceutical Sciences, Fukuoka University, 8-19-1 Nanakuma, Jonan- ku, Fukuoka, 814-0180 Japan; 2 Department of Pharmacy, Fukuoka University Hospital, 7-45-1 Nanakuma, Jonan-ku, Fukuoka, 814-0180 Japan; 3 Department of Surgery, Fukuoka University Chikushi Hospital, 1-1-1, Zokumyoin, Chikushino-shi, Fukuoka, 818-8502 Japan; 4 Department of Pharmacy, Fukuoka University Chikushi Hospital, 1-1-1, Zokumyoin, Chikushino-shi, Fukuoka, 818-8502 Japan

**Keywords:** Syndrome of inappropriate antidiuretic hormone secretion, Hyponatremia, Oxaliplatin, Capecitabine, Bevacizumab, Colon cancer

## Abstract

**Background:**

Hyponatremia is an electrolyte abnormality that is often caused by the syndrome of inappropriate antidiuretic hormone secretion (SIADH) and frequently encountered in the field of oncology. Although SIADH is a known complication of certain chemotherapeutic agents, its occurrence with oxaliplatin is rare. We report a case of SIADH in a patient with colon cancer who was undergoing treatment comprising capecitabine and oxaliplatin (CAPOX) plus bevacizumab.

**Case presentation:**

A 70-year-old man with stage cT4bN2M0 colon cancer underwent chemotherapy with CAPOX plus bevacizumab. On day 7 of treatment, the patient developed severe hyponatremia (serum sodium level, 108 mmol/L) accompanied by nausea and ileus. Laboratory test results were consistent with SIADH, including low serum osmolality, elevated urine osmolality, elevated sodium concentration, and elevated antidiuretic hormone levels. The condition improved with 3% saline infusion and fluid restriction. No other underlying causes, such as central nervous system lesions or adrenal or thyroid dysfunction, were identified. CAPOX-induced SIADH was diagnosed based on clinical findings and the exclusion of other etiologies. Transition to second-line therapy was performed without SIADH recurrence.

**Conclusions:**

Oxaliplatin-based regimens may rarely induce SIADH. Clinicians should be vigilant of electrolyte disturbances during chemotherapy and promptly manage hyponatremia to avoid severe complications.

## Background

Hyponatremia, which is typically defined as a serum sodium level less than 135 mmol/L, is a common electrolyte abnormality encountered in oncology practice [1, 2]. Severe hyponatremia, which is characterized by a serum sodium level less than 120 mmol/L, is a critical electrolyte disorder that can lead to life-threatening neurological complications. The syndrome of inappropriate antidiuretic hormone secretion (SIADH) is the leading cause of hyponatremia in patients with cancer; furthermore, it affects approximately 1–2% of all patients with cancer and accounts for approximately 30% of all hyponatremia cases [3]. With SIADH, hyponatremia occurs without alterations in the circulating blood volume and reflects the relative ratio of sodium to blood water. SIADH can be triggered by various factors such as central nervous system diseases, pulmonary diseases, ectopic vasopressin-producing tumors, and drug use. Additionally, SIADH has been associated with various anticancer drug regimens, including cytotoxic agents such as vinca alkaloids, platinum compounds, and alkylating agents [4]. Among platinum compounds, cisplatin is more frequently associated with hyponatremia than carboplatin. Previous studies have indicated that mechanisms such as SIADH and renal salt wasting contribute to hyponatremia after cisplatin administration [5, 6]. To date, only one case of SIADH associated with oxaliplatin has been reported [7]. We describe a case of SIADH caused by the administration of capecitabine and oxaliplatin (CAPOX) plus bevacizumab in a patient with colon cancer.

## Case presentation

A 70-year-old man presented with abdominal pain and underwent abdominal ultrasonography that revealed a mass on the lateral side of the right kidney. Lower gastrointestinal endoscopy revealed an ulcerative and localized neoplastic lesion in the ascending colon. Moderately to poorly differentiated adenocarcinoma was diagnosed based on the biopsy results, and the patient was referred to our hospital. Computed tomography (CT) showed infiltration into the abdominal wall and right kidney as well as multiple mesenteric lymph nodes; therefore, clinical stage cT4bN2M0 was finally diagnosed. Prior to treatment, the levels of carcinoembryonic antigen (CEA) and carbohydrate antigen 19 − 9 (CA19-9), which are tumor markers, were 2.1 ng/mL and 7 U/mL, respectively. The patient’s medical history included gastric ulcer, hypertension, and cerebral infarction, and his current medications included clopidogrel, valsartan, ifenprodil, folic acid, and teprenone. The patient had no history of drug or food allergies.

**Fig. 1:**
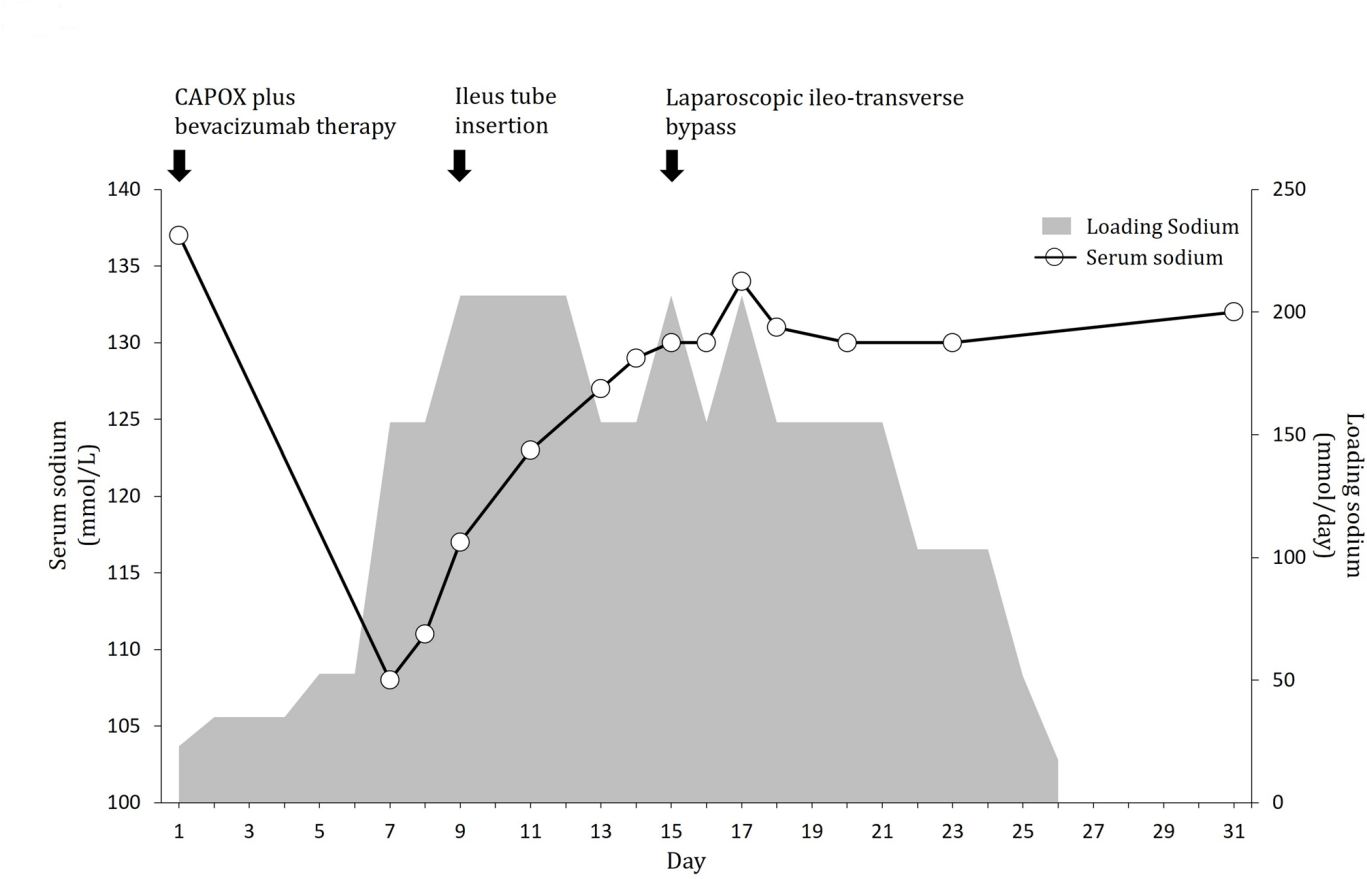
Clinical course of the patient after capecitabine and oxaliplatin (CAPOX) plus bevacizumab therapy

The clinical course of this case is shown in Fig. 1. Systemic chemotherapy comprising CAPOX plus bevacizumab (oxaliplatin 130 mg/m^2^ and bevacizumab 10 mg/kg) was administered on day 1, followed by oral capecitabine 2000 mg/m^2^ twice daily beginning from the evening of day 1 until the morning of day 15; this regimen was repeated every 3 weeks. Antiemetic prophylaxis included intravenous palonosetron (a 5-hydroxytryptamine 3 [5-HT_3_] receptor antagonist; 0.75 mg) and dexamethasone (4.95 mg) administered 30 min before oxaliplatin on day 1. Additionally, oral aprepitant (a neurokinin 1 [NK_1_] receptor antagonist; 125 mg) was administered up to 1 h before oxaliplatin administration on day 1. On days 2 and 3, oral aprepitant (80 mg) was administered after breakfast. On day 2, the patient presented with grade 2 hiccups, grade 3 nausea, and grade 3 anorexia; therefore, a 1000-mL electrolyte infusion was initiated. On day 3, the patient experienced difficulty with capecitabine. On day 7, laboratory tests revealed that the serum sodium level decreased from 137 to 108 mmol/L (reference: 138–145 mmol/L). The patient was referred to the Department of Endocrinology for a detailed examination. The urine osmolality was 746 mOsm/kg (reference: 50–1300 mOsm/kg), serum osmolality was 229 mOsm/L (reference: 276–292 mOsm/L), urinary sodium concentration was 128 mmol/L, and plasma antidiuretic hormone was 3.1 pg/mL. Additional laboratory results are presented in Table 1. To correct hyponatremia, an intravenous infusion of 3% saline was initiated at a rate of 0.5 to 1 mL/kg/hour on day 7. On day 8, the patient developed worsening abdominal distension and experienced the absence of bowel movements for 7 days as well as episodes of vomiting. Abdominal radiography revealed significant gas accumulation and dilatation in the small intestine that required nasogastric tube insertion for decompression. On day 9, computed tomography suggested ileal invasion by the tumor. Despite intervention, nausea and fatigue persisted, prompting the insertion of a decompression tube to relieve the suspected ileus. On day 10, the patient experienced a large bowel movement. The serum sodium level gradually improved with a 3% saline infusion and reached 129 mmol/L on day 14. We diagnosed SIADH induced by CAPOX plus bevacizumab because head and chest computed tomography indicated no abnormalities. No evidence of adrenal, thyroid, cardiac, renal, or liver dysfunction was observed. Clinical euvolemia, normal blood pressure, absence of peripheral edema, and no signs of thirst or dry mucous membranes were noted. On day 15, the patient underwent laparoscopic ileotransverse colon bypass surgery for persistent bowel obstruction. Postoperatively, sodium supplementation was gradually tapered according to the patient’s condition. Throughout this period, the serum sodium level remained stable and within the range of 130 to 134 mmol/L. The patient was discharged on day 31. On day 46, second-line chemotherapy comprising the SIRB regimen (bevacizumab, irinotecan, and S-1) was initiated. SIADH recurrence was not observed during the course of treatment.

**Table 1:** Laboratory results before CAPOX plus bevacizumab therapy and on day 7

Investigation	Before CAPOX plus bevacizumab therapy	Day 7	Normal range
Body weight (kg)	45.8	46.0	−
Serum sodium (mmol/L)	137	108	138–145
Serum potassium (mmol/L)	4.6	4.1	3.6–4.8
Serum creatinine (mg/dL)	0.91	0.52	0.65–1.07
Serum glucose (mmol/L)	101	119	73–109
Serum osmolality (mOsm/kg)	−	229	276–292
Urine osmolality (mOsm/kg)	−	746	−
Urine sodium (mmol/L)	−	128	−
Morning cortisol (µg/dL)	−	19.9	6.24–18
Plasma ADH (pg/mL)	−	3.1	≤ 2.8
Plasma renin activity (ng/ml/h)	−	2.3	0.3–2.9
Plasma ACTH (pg/mL)	−	34.6	7.2–66.3

ADH, antidiuretic hormone, ACTH, adrenocorticotropic hormone; CAPOX, capecitabine and oxaliplatin

## Discussion and Conclusions

SIADH is a condition in which the antidiuretic hormone action of vasopressin persists despite hyponatremia. The body fluid volume is normal; therefore, when determining the diagnosis, SIADH must be differentiated from diseases that are accompanied by dehydration or an increased extracellular fluid volume. If the progression of hyponatremia is slow, then it often does not cause any symptoms; however, if hyponatremia is severe or the progression is rapid, then symptoms such as fatigue, loss of appetite, and impaired consciousness may appear. For cases of hyponatremia accompanied by impaired consciousness, serum sodium was corrected by administering a 3% saline solution. However, careful management is required because a rapid increase in the serum sodium concentration may cause osmotic demyelination syndrome.

SIADH is a common electrolyte abnormality associated with advanced cancer. The prevalence of SIADH among patients receiving chemotherapy is 3.7% [4, 8]. Chemotherapeutic agents that induce SIADH include cisplatin, carboplatin, vincristine, cyclophosphamide, and certain molecular-targeted drugs. SIADH may be triggered by chemotherapy-related side effects such as vomiting, diarrhea, or large-volume fluid infusions, leading to electrolyte imbalances [4, 8]. As hyponatremia caused by SIADH progresses, it can result in symptoms such as loss of appetite, nausea, confusion, convulsions, and coma. Therefore, effective management of hyponatremia is essential to cancer treatment.

The diagnostic criteria for SIADH published by the Japanese Endocrine Society include the following: absence of dehydration; serum sodium concentration less than 135 mmol/L; plasma osmolality less than 280 mOsm/kg; nonsuppressed plasma vasopressin concentration despite hyponatremia and hypoosmolality; urine osmolality more than 100 mOsm/kg; urinary sodium concentration more than 20 mmol/L; and normal renal, adrenal, and thyroid functions [9]. In healthy individuals, dehydration promotes the secretion of antidiuretic hormone (ADH), leading to water retention; however, water overload inhibits ADH secretion, promoting diuresis. Therefore, the normal reference range for vasopressin is 4.0 pg/mL or less under water restriction and 2.8 pg/mL or less under free water intake. As plasma ADH is present in very low concentrations, it is necessary to interpret the results in conjunction with plasma osmolarity. In this case, plasma osmolarity was 229 mOsm/kg, whereas plasma ADH was elevated at 3.1 pg/mL. This case met al.l the aforementioned criteria and was diagnosed as SIADH by an endocrinologist.

Hyponatremia caused by cisplatin, a platinum compound, is often attributed to either SIADH or renal salt-wasting syndrome caused by dehydration and renal dysfunction [10]. However, reports of SIADH associated with oxaliplatin are rare, and the underlying mechanisms remain unclear. In this case, the Naranjo Adverse Event Causality Scale [11] indicated a score of 4, suggesting a “possible” relationship between the adverse event and suspected drug (Table 2). Due to the simultaneous occurrence of ileus, stress, and sodium loss, which are known triggers of SIADH, the possibility of multifactorial etiology cannot be ruled out. The differential diagnosis included cerebral salt-wasting syndrome; however, this condition was deemed unlikely based on the clinical findings. Throughout the disease course, the patient showed no signs of hypovolemia, such as weight loss, decreased skin turgor, or orthostatic hypotension. His blood pressure remained within the normal range, and his hematocrit level was 38.6%, without any indication of hemoconcentration. Furthermore, imaging studies revealed no evidence of intracranial abnormalities. Although serum uric acid levels and the fractional excretion of uric acid were not measured, the overall clinical picture did not support a diagnosis of cerebral salt-wasting syndrome. With paraneoplastic SIADH, hyponatremia is present before the start of treatment, and serum sodium levels improve with treatment. However, in this case, hyponatremia developed after the start of chemotherapy; therefore, we concluded that the possibility of tumor-associated SIADH was low.

**Table 2:** Calculation of the probability of an adverse drug reaction occurring in the patient based on the Naranjo adverse drug reaction probability scale

		Yes	No	Unknown	Score
1.	Are there previous conclusive reports of this reaction?	+ 1	0	0	1
2.	Did the adverse event appear after the suspected drug was administered?	+ 2	−1	0	2
3.	Did the adverse reaction improve when the drug was discontinued or a specific antagonist was administered?	+ 1	0	0	1
4.	Did the adverse reaction reappear when the drug was readministered?	+ 2	−1	0	0
5.	Are there alternative causes (other than the drug) that could on their own have caused the reaction?	−1	+ 2	0	-1
6.	Did the reaction reappear when a placebo was given?	−1	+ 1	0	0
7.	Was the drug detected in the blood (or other fluids) in concentrations known to be toxic?	+ 1	0	0	0
8.	Was the reaction more severe when the dose was increased or less severe when the dose was decreased?	+ 1	0	0	0
9.	Did the patient have a similar reaction to the same or similar drugs in any previous exposure?	+ 1	0	0	0
10.	Was the adverse event confirmed by any objective evidence?	+ 1	0	0	1
		Total Score		4	

Total scores ranged from − 4 to + 13. The reaction was considered definite if the score was ≥ 9, probable if the score was 5 to 8, possible if the score was 1 to 4, and doubtful if 0 or less

No explicit mention of SIADH was observed during a review of the package inserts of concomitant medications. Although hyponatremia is a known adverse effect of clopidogrel and valsartan, the reported incidence of SIADH with both these drugs is less than 1%. Additionally, as of August 2024, fewer than five cases of SIADH associated with oxaliplatin or capecitabine have been reported in the Japanese Adverse Drug Event Report database.

The patient showed no apparent signs of dehydration or renal dysfunction. However, the development of ileus on day 8 may have contributed to SIADH through sodium loss from the gastrointestinal tract caused by vomiting. In addition, cancer-related factors such as anticancer drug therapy, surgery, pain, and nausea are known to increase vasopressin secretion, predisposing patients to hyponatremia [3]. In this case, nausea and appetite loss likely contributed to the onset of SIADH. Notably, bevacizumab was administered again during second-line chemotherapy without SIADH recurrence; therefore, it was an unlikely causative agent. Previous reports of SIADH have been limited to SOX therapy (S-1 and oxaliplatin); importantly, SIADH recurrence has not been observed with re-administration of S-1 alone [7]. Moreover, Futai et al. reported a case of SIADH that occurred following cisplatin and 5-fluorouracil (5-FU) therapy; however, after switching to a regimen of folinic acid, 5-FU, and oxaliplatin (FOLFOX), SIADH recurrence was not observed [12]. These findings suggest that 5-FU–based agents, including capecitabine, are less likely to be the primary agents that cause SIADH. In this case, an S-1–containing regimen was administered as second-line treatment, but SIADH did not recur. Although a definitive conclusion cannot be drawn because of the limited number of cases, based on the available literature and mechanistic plausibility, we considered oxaliplatin as a more plausible causative agent than capecitabine in our case. The patient discontinued the treatment regimen after one course of CAPOX; therefore, therapy was transitioned to second-line treatment. As neither oxaliplatin nor capecitabine was readministered, determining the exact causative drug was challenging.

Compared with the previously reported case of SIADH induced by oxaliplatin-based chemotherapy in a patient with gastric cancer [7], our case comprised several novel aspects that underscore its educational and clinical value. First, the underlying malignancy in our case was colon cancer. Second, although the previous case involved the SOX regimen (S-1 and oxaliplatin), our patient was treated with the CAPOX regimen combined with bevacizumab; therefore, this is the first reported case of SIADH associated with CAPOX plus bevacizumab therapy. Third, the clinical presentation was complicated by ileus, which likely exacerbated the sodium imbalance through vomiting and gastrointestinal sodium loss. This complexity adds valuable insight regarding the multifactorial nature of SIADH in oncology settings. These distinctions highlight the importance of individualized evaluations of patients receiving oxaliplatin-containing regimens and reinforce the need for awareness of SIADH in diverse clinical contexts.

Although oxaliplatin is associated with a lower risk of SIADH compared to that associated with cisplatin or carboplatin, it is a potential but rare cause of SIADH. This case highlights the importance of recognizing SIADH as a potential complication of oxaliplatin-based chemotherapy and emphasizes the need for vigilance during the management of electrolyte imbalance in patients with cancer.

## Data Availability

No datasets were generated or analysed during the current study.

## Abbreviations

ADH: antidiuretic hormone secretion
CAPOX: capecitabine and oxaliplatin
CA19-9: carbohydrate antigen 19 − 9
CEA: carcinoembryonic antigen
CT: computed tomography
NK_1_: neurokinin 1
SIADH: syndrome of inappropriate antidiuretic hormone secretion
5-HT_3_: 5-hydroxytryptamine 3
